# Somatic Blood Mosaicism and Psychiatric Disease: Current Evidence and Causal Limits

**DOI:** 10.3390/genes17101224

**Published:** 2026-10-02

**Authors:** Andrada-Adelaida Belbe, Lorin-Manuel Pîrlog, Alissia-Nicoleta Pilatec, Mariela-Sanda Militaru, Irina-Ioana Iordănescu, Andreea Cătană

**Affiliations:** 1 Department of Molecular Sciences, Faculty of Medicine, “Iuliu Hațieganu” University of Medicine and Pharmacy, 400012 Cluj-Napoca, Romania; adelaidapatrascanu@gmail.com (A.-A.B.); pilatecalissia@gmail.com (A.-N.P.); sanda.militaru@umfcluj.ro (M.-S.M.); catanaandreea@elearn.umfcluj.ro (A.C.); 2 Department of Medical Genetics, Clinical Emergency Hospital for Children, 400370 Cluj-Napoca, Romania; 3 Regional Laboratory Cluj-Napoca, Department of Medical Genetics, Regina Maria Health Private Network, 400363 Cluj-Napoca, Romania; 4 Genetic Centre Laboratory, Department of Medical Genetics, Regina Maria Health Private Network, 011376 Bucharest, Romania; irina.iordanescu@reginamaria.ro; 5 Genetics Department, “Carol Davila” University of Medicine and Pharmacy, 020027 Bucharest, Romania; 6 Department of Oncogenetics, “Prof. Dr. I. Chiricuță” Institute of Oncology, 400015 Cluj-Napoca, Romania

**Keywords:** clonal haematopoiesis, CHIP, somatic mosaicism, mosaic chromosomal alterations, developmental mosaicism, schizophrenia, bipolar disorder, depression, substance-use disorder, neuroinflammation

## Abstract

Somatic blood mosaicism is an established feature of ageing and a heterogeneous marker of haematologic cancer and inflammatory and vascular risk, but its psychiatric evidence base remains small. This structured critical narrative review evaluates mutation-defined clonal haematopoiesis, clonal haematopoiesis of indeterminate potential (CHIP), autosomal mosaic chromosomal alterations (mCAs), and sex-chromosome loss while separating probable developmental mosaicism from adult-acquired blood clonality. Direct human evidence comprises seven methodologically heterogeneous studies. Psychiatric CHIP findings rely mainly on one prospective UK Biobank cohort of 360,451 participants, in which CHIP was associated with any incident psychiatric disorder (hazard ratio 1.15), particularly major depressive and substance-use disorders. Separate studies report excess mosaic losses in schizophrenia and bipolar disorder and autosomal or X-chromosome mosaicism in suicide. Clone-size gradients and lesion-specific findings add biological structure, but do not provide independent replication. Most mechanistic support comes from cardiovascular, stroke and neurodegenerative research: mutant myeloid cells can alter inflammasome signalling, endothelial function, thrombosis and tissue macrophage behaviour. These pathways make indirect brain effects plausible without establishing a psychiatric mechanism. Blood detection also cannot date a mosaic event or demonstrate the same clone in neural tissue. Taken together, the available evidence supports somatic blood mosaicism as a potentially informative marker of psychiatric heterogeneity and shared inflammatory, vascular and ageing-related biology, but does not establish a causal blood-clone-to-brain pathway. Routine psychiatric screening for CHIP or mCAs is therefore not currently justified. The immediate research priority is independent replication in longitudinal cohorts with repeated sequencing, detailed exposure and treatment histories, and direct testing of inflammatory, vascular and neuroimaging intermediates capable of distinguishing cause, consequence and shared risk.

## 1. Introduction

Post-zygotic variants accumulate in self-renewing tissues, and selected variants can generate detectable clonal populations. Blood is the best-characterized compartment because it is accessible, continuously renewed and represented in large sequencing biobanks. Population studies established that apparently healthy carriers of leukemia-associated somatic variants have increased risks of haematologic malignancy and all-cause mortality, with much of the excess mortality linked to cardiovascular disease. Clonality is therefore a premalignant and systemic ageing state, not an inevitable precursor of leukaemia; absolute risk varies markedly by driver, clone size, co-mutations, cytopenias and clonal growth [1,2,3,4,5,6,7,8,9,10,11,12].

Direct psychiatric evidence is recent and limited. One large prospective cohort linked CHIP with incident psychiatric disorders, principally major depressive and substance-use disorders [13]. Separate case–control studies reported mosaic chromosomal losses in schizophrenia and bipolar disorder and autosomal or X-chromosome mosaicism in suicide [14,15,16]. These studies differ in lesion class, phenotype, population and ascertainment, so they do not establish a common psychiatric clonality signature.

Mosaic loss of the Y chromosome (mLOY) is a related but distinct exposure. Blood mLOY findings in suicide and schizophrenia are inconsistent, and one post-mortem study did not detect the blood association in dorsolateral prefrontal cortex [17,18,19]. Tissue, age, smoking, assay method and clinical course therefore remain central to interpretation.

This review asks what the current data permit us to infer about somatic blood mosaicism and psychiatric disease. Four models remain plausible: blood clonality contributes to psychiatric outcomes through systemic intermediates; psychiatric illness or correlated exposures select or expand clones; shared causes generate both; or feedback operates in both directions. These models make different predictions about temporal order, clone growth and mediation, and they are evaluated in Section 5 [20,21,22,23].

The review’s contribution is a psychiatric synthesis that keeps lesion classes and developmental timing separate. Probable developmental mosaicism may involve blood and brain, whereas adult-acquired clonal haematopoiesis is usually blood restricted and would be expected to act indirectly. The reverse clinical questionwhether psychiatric illness modifies haematologic, oncologic or vascular outcomes among clone carriersis considered in parallel.

A structured literature search was conducted in PubMed, Scopus, Web of Science and Google Scholar and was last updated on 12 September 2026. Searches combined terms related to clonal haematopoiesis, clonal haematopoiesis of indeterminate potential (CHIP), somatic mosaicism, mosaic chromosomal alterations (mCAs), sex-chromosome loss and developmental mosaicism with psychiatric and neuropsychiatric terms including depression, schizophrenia, bipolar disorder, suicide, substance-use disorders and neuropsychiatric phenotypes. Additional targeted searches addressed inflammation, white-matter and neuroimaging phenotypes, blood–brain barrier biology, vascular disease, cancer, cytotoxic therapy and clonal evolution.

This is a critical narrative review rather than a systematic review or meta-analysis. Study selection was therefore interpretive rather than exhaustive. Priority was given to direct human psychiatric studies, longitudinal or temporally informative designs, lesion- or clone-size-resolved analyses, and experimental studies directly examining biologically plausible systemic mechanisms. Direct psychiatric evidence was evaluated separately from mechanistic evidence derived from other disease settings, and heterogeneous lesion classes, assays and outcomes were not quantitatively pooled. Pregnancy-acquired fetal microchimerism was excluded because it represents persistence of genetically distinct fetal cells rather than post-zygotic variation within the host [2,23].

This review also has methodological limitations. As a critical narrative review, it was not conducted according to a preregistered systematic-review protocol, and formal risk-of-bias assessment or quantitative evidence synthesis was not undertaken. Study selection was interpretive rather than exhaustive, and some relevant publications may therefore have been missed. In addition, the mechanistic literature was selected to illustrate biologically plausible pathways rather than to provide a comprehensive review of clonal haematopoiesis across cardiovascular, neurological and inflammatory disease. These limitations should be considered when interpreting the scope and strength of the conclusions.

## 2. Definitions and Biological Boundaries

### 2.1. Clonal Haematopoiesis CHIP and Related States

**Figure 1:**
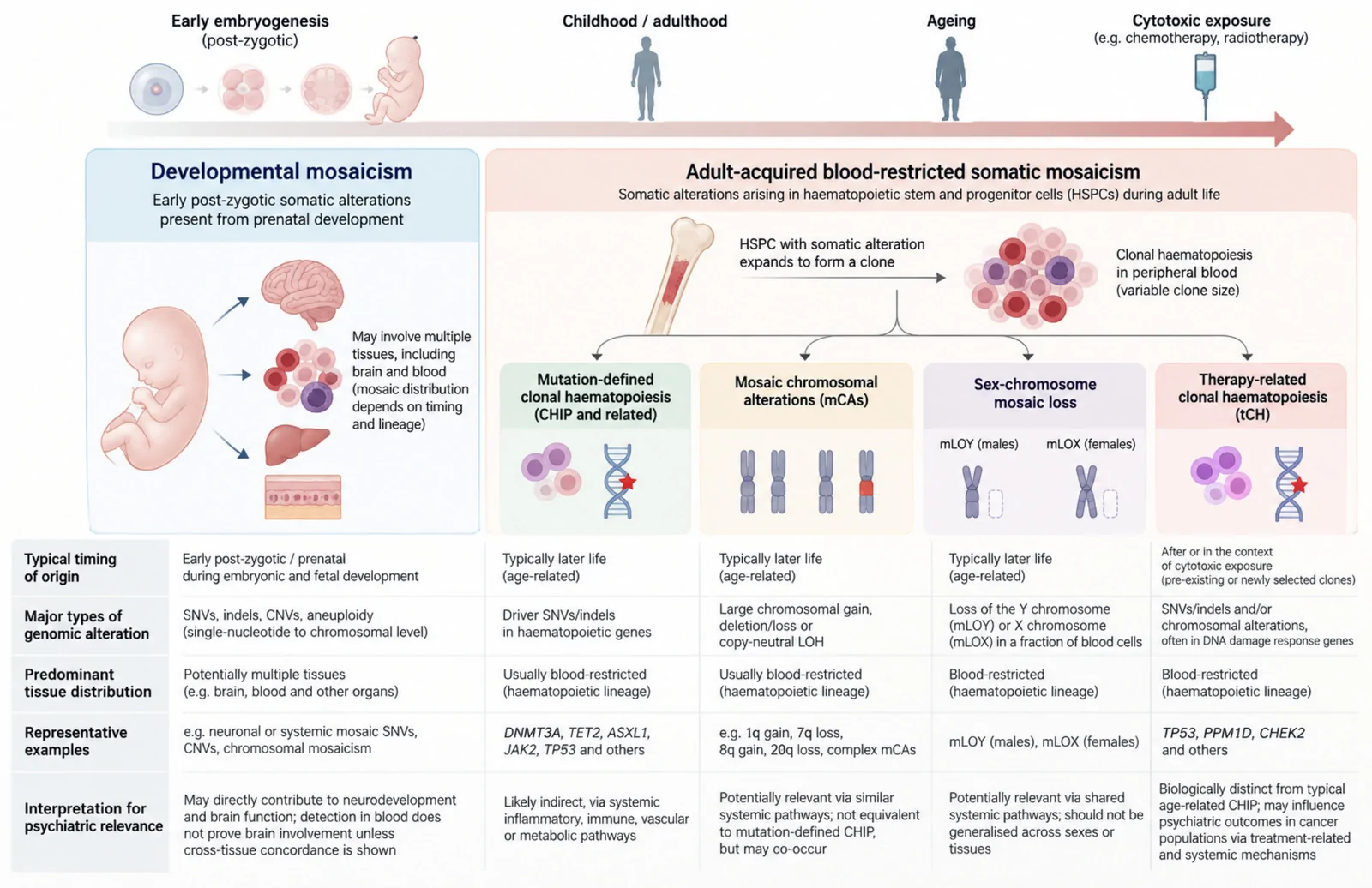
Conceptual framework of somatic mosaicism relevant to psychiatric disease. The schematic distinguishes developmental mosaicism from predominantly adult-acquired, blood-restricted somatic mosaicism according to timing, genomic alteration, and tissue distribution. Developmental events may involve both blood and brain, whereas adult-acquired clonal haematopoiesis, mCAs, sex-chromosome loss, and therapy-related clones are generally confined to haematopoietic lineages and would be expected to influence psychiatric phenotypes indirectly. Blood detection alone does not establish developmental timing or neural involvement. Red stars indicate acquired somatic mutations.

Clonal haematopoiesis is the expansion of blood cells descended from one haematopoietic stem or progenitor cell. CHIP conventionally denotes a recognized myeloid-neoplasm driver, often at a variant allele fraction (VAF) of at least 2%, without an overt haematologic malignancy or qualifying cytopenia. This threshold is historical and assay dependent. CHIP must also be distinguished from clonal cytopenia of undetermined significance, overt myeloid disease, and lymphoid clonal haematopoiesis because these states carry different baseline risks and trajectories [24,25,26,27,28,29]. See Table 1. The principal forms of somatic mosaicism discussed in this review and their differences in timing, genomic alteration, and tissue distribution are summarized in Figure 1.

### 2.2. Driver Identity and Clonal Architecture

Population CHIP is dominated by *DNMT3A*, *TET2* and *ASXL1*, but driver genes are not interchangeable. *JAK2* p.V617F is strongly thrombo-inflammatory; *TP53* and *PPM1D* clones are preferentially selected by DNA damage; and spliceosome or selected transcription-factor mutations can carry disproportionate malignant risk [36,37,38].

Bulk-blood VAF does not identify the mature immune compartment carrying a variant, and clonal architecture changes over time. Clone size, mutation number, lineage distribution and growth rate add information beyond binary CHIP status. Risk models that integrate molecular features, blood counts and age sharply separate low- and high-risk carriers [5,6,28,39,40,41,42,43,44]. Psychiatric studies should therefore report sequencing depth, driver identity and clonal architecture rather than CHIP prevalence alone. See Table 1.

### 2.3. Mosaic Chromosomal Alterations

mCAs are large, acquired gains, deletions or copy-neutral loss-of-heterozygosity events detected from allelic imbalance and intensity patterns on SNP arrays or sequencing. They partly overlap mutation-defined CHIP but are not equivalent to it. Array density, phasing, coverage, filtering and minimum cell fraction affect detection, especially in younger samples where events are rare. Odds ratios from studies using different platforms and thresholds should not be treated as estimates of one exposure [14,15,16,30,31,45,46,47,48,49,50].

Age, sex, ancestry and germline susceptibility shape mCA distribution and clonal fitness. mLOY is a sex-specific case strongly related to age, inherited susceptibility and smoking, and it cannot be generalized to autosomal mosaic loss or to women. Organ-specific effects, biomarker changes and infection associations make systemic consequences plausible, but their direction depends on chromosome, lineage and clone size [31,46,47,48,49,50,51,52,53,54,55,56,57,58,59,60]. See Table 1.

### 2.4. Developmental Versus Age-Related Mosaicism

An early post-zygotic mutation may populate several germ layers and reach both blood and brain. Adult-acquired clonal haematopoiesis, by contrast, is usually confined to haematopoietic lineages. Human forebrain and neuronal studies show that developmental clones can reveal lineage history and, in some cases, affect neurodevelopment, but a variant detected in blood does not establish developmental timing, multi-tissue distribution or neural involvement [32,33,61,62,63].

This distinction is decisive for schizophrenia and bipolar disorder. A prenatal neural clone could act directly on neurodevelopment, whereas adult blood clonality would require immune, vascular, metabolic or endocrine mediation. Somatic mutational signatures are characteristic patterns of acquired DNA changes that can provide clues to the biological processes responsible for generating those mutations. In schizophrenia brain tissue, such patterns have been interpreted as evidence of prenatal mutational activity, but they do not establish a mechanism for age-related blood CHIP. Multi-tissue sequencing, phylogenetic reconstruction, lineage analysis and repeated sampling are needed to distinguish developmental mosaicism, adult haematopoietic clonality, sex-chromosome loss and treatment-selected clones [14,15,16,17,18,19,20,31,32,33,34,35,36,37,38,46,47,48,49,50,51,52,53,54,55,61,62,63]. See Table 1.

### 2.5. Cancer Relevance and Treatment Selection

CHIP and mCAs are premalignant substrates, not inevitable leukemia. Transformation risk is concentrated in particular genotypes, larger or expanding clones, multiple mutations and cytopenias; absolute risk therefore varies by orders of magnitude [5,6,7,8,9,10,11,12].

Cancer matters here mainly as a boundary condition and source of bias. Blood-derived variants can be misattributed in plasma or unpaired tumour sequencing, while chemotherapy and radiotherapy preferentially select DNA-damage-response clones such as *TP53*, *PPM1D* and *CHEK2*. A treatment-selected clone is not comparable to a small age-related *DNMT3A* clone. Psychiatric studies should capture cancer history, treatment type and timing and use paired blood-tumour data when somatic origin is uncertain. Several practical scenarios illustrate this problem. First, patients with cancer may have a higher prevalence of therapy-selected clonal haematopoiesis, particularly involving DNA-damage-response genes such as *TP53* and *PPM1D*, after chemotherapy or radiotherapy [34,35,64,65,66,67,68,69]. These clones may therefore reflect treatment history rather than the same biology as age-related *DNMT3A*- or *TET2*-associated clonality. Second, cancer survivors may also have higher burdens of depression, fatigue, pain, vascular disease, and medication exposure, creating potential confounding between clonal alterations and psychiatric outcomes. Third, blood-derived clonal variants can be detected in plasma or unpaired tumour sequencing and may be misclassified as tumour-derived alterations if a matched normal sample is unavailable [66,67]. Together, these examples show how cancer diagnosis, treatment, survivorship, and assay design can influence both the prevalence and interpretation of somatic blood mutations and thereby distort apparent associations with psychiatric phenotypes [34,35,64,65,66,67,68,69,70,71,72,73,74,75,76].

## 3. Direct Human Psychiatric Evidence

The seven direct human studies examine different psychiatric outcomes and different forms of mosaicism. Depression, substance-use disorders, schizophrenia, bipolar disorder, suicide and neuroimaging traits should be interpreted by lesion class and design rather than combined into one evidence estimate [13,14,15,16,17,18,19]. See Table 2.

### 3.1. Major Depressive and Substance Use Disorders

The only large prospective psychiatric CHIP analysis included 360,451 UK Biobank participants without recorded psychiatric diagnoses at baseline. The main Results section reports a median follow-up of 13.1 years. CHIP was associated with any incident psychiatric disorder (HR 1.15), with statistically supported associations for major depressive disorder (HR 1.12) and substance-use disorders (HR 1.27). Larger clones and gene-specific estimates showed internal heterogeneity, but all psychiatric CHIP findings come from this single discovery cohort [13].

These effect sizes are modest. Associations of approximately 1.1–1.3 may matter at population scale, but residual confounding of similar magnitude could account for them, and carrier status does not imply that an individual will develop psychiatric disease. Absolute risk, age-specific incidence and competing mortality remain necessary for prediction [13,77].

The same study linked CHIP, especially *DNMT3A*-CHIP, with 11 white-matter imaging-derived phenotypes. This does not supply independent replication: the imaging subset was smaller, mediation was not tested, and late-life incident diagnoses may reflect vascular or cognitive pathways that differ from early-onset mood disease [13,78,79].

### 3.2. Schizophrenia and Bipolar Disorder

In 9715 Chinese schizophrenia cases and 28,822 controls, filtered blood-derived somatic CNV losses were more frequent in cases (1.00% versus 0.52%; OR 1.91, 95% CI 1.47–2.49), with larger estimates at higher cell fractions. A European Psychiatric Genomics Consortium dataset provided cross-cohort support, but a separate post-mortem brain analysis showed only limited locus-level overlap. No study demonstrated the same clone in blood and brain from the same person, and early developmental timing remains inferred [14,80].

A later study of 2470 schizophrenia cases, 3732 bipolar disorder cases and 177,773 controls found more autosomal mCAs, particularly losses, in schizophrenia (OR 1.78) and bipolar disorder (OR 1.41). Young-age subgroup estimates were much larger (OR 7.01 and 4.01), and effects rose with cell fraction. These results are provocative but vulnerable to sparse events, age subgrouping, survivor and biobank selection, and unresolved timing of clone acquisition [16].

Smaller mLOY studies are inconsistent: one found no case–control difference but an association with illness duration, while another found a higher mosaic fraction without a higher prevalence. Illness duration, age at onset and age at sampling are intertwined, making reverse causation or cumulative exposure at least as plausible as a causal Y-loss model. The convergence of larger autosomal studies on mosaic loss is notable, but clinically recruited ancestry-diverse cohorts with serial samples are still needed [14,15,16,18,19].

### 3.3. Suicide

Across two case–control cohorts totalling 773 suicide decedents and 177,740 controls, autosomal mCAs were associated with suicide death (OR 1.78), with larger estimates for losses (OR 2.70) and gains (OR 2.23); mLOX was enriched among women (OR 4.00). Male mLOY showed an inverse estimate (OR 0.71, *p* = 0.0256) that did not meet the prespecified Bonferroni threshold of *p* < 0.00833 and should be reported as a suggestive trend, not a confirmed association [15].

An earlier Japanese study reported more blood mLOY in male suicide completers but no corresponding association in dorsolateral prefrontal cortex. The discordant mLOY results reinforce that blood mosaicism is not a proxy for neural mosaicism and that age, smoking, assay and population structure can change the observed association. Suicide is also a transdiagnostic outcome rather than a single psychiatric disorder [15,17].

### 3.4. Evidence Hierarchy and Current Limits

Evidence maturity differs by phenotype and lesion class. Depression and substance-use findings rest on one prospective CHIP cohort. Schizophrenia has several somatic-loss datasets, but they use different assumptions about timing and tissue distribution. Bipolar evidence is newer, and suicide findings are case–control with discordant sex-chromosome results [13,14,15,16,17,18,19].

Larger clones or higher cell fractions often show stronger associations. Such gradients add internal biological structure, but they are not independent replication and do not prove causality because clone burden also reflects age, duration and selective fitness. Gene-specific estimates require validation before they can define a psychiatric molecular subtype [13,14,16,81].

Point-mutation CHIP, autosomal mCAs, mLOY or mLOX, and probable developmental CNVs should remain separate exposures until harmonized cohorts show convergence. Shared gene names also do not establish a shared cellular mechanism: a *DNMT3A* or *TET2* variant confined to blood does not alter neuronal genotype. Direct cross-tissue claims require the variant, relevant cell type and temporally appropriate function to be demonstrated in neural tissue [28,31,37,43,55,63,82].

## 4. Biological Plausibility Outside Psychiatry

Experimental studies of clonal haematopoiesis establish that a minority blood clone can causally affect distant organs without malignant transformation in non-psychiatric disease models. For example, *Tet2*-deficient haematopoiesis can accelerate atherosclerosis and heart failure through macrophage and inflammasome pathways. These findings provide causal evidence for systemic effects of clonal haematopoiesis in those experimental settings and support biological plausibility for indirect effects on the brain, but they do not constitute causal evidence for psychiatric disease [83,84,85].

Mechanisms vary by driver. IL-1 and IL-6 pathways, AIM2, BRCC3-NLRP3 signalling, and thrombo-inflammatory meprogrammes differ across *TET2*-, *DNMT3A*-, *TP53*-, *ASXL1*- and *JAK2*-associated states. Psychiatric studies identify inflammatory subgroups, but they do not show that CH is the source of inflammation or that cardiovascular anti-inflammatory results predict psychiatric treatment response [86,87,88,89,90,91,92,93,94,95,96,97,98].

Conversely, ageing, obesity, inflammation, sleep disruption and related exposures may alter haematopoietic stem-cell fitness, making reverse causation and bidirectional feedback biologically plausible [99,100,101,102].

A vascular intermediary may be as plausible as direct cytokine-to-neuron signalling. CH is associated with cardiovascular disease and stroke, and mutant myeloid cells can alter endothelial and thrombotic biology. Cerebrovascular injury could then influence mood, cognition and white matter. Yet experimental effects can oppose one another: *DNMT3A* dysfunction worsened acute neuroinflammation in one stroke model, whereas *Tet2*-mediated CH modestly improved subacute recovery in another [83,103,104,105,106,107,108,109].

Human imaging findings are not uniformly adverse. The UK Biobank psychiatric study associated CHIP with white-matter measures, while other cohorts reported covert cerebral changes or lower white-matter hyperintensity volume with *DNMT3A* clones. Blood–brain barrier and CNS-myeloid evidence comes largely from stress, neurodegenerative and mouse studies, where clone effects can be harmful or protective [13,78,79,110,111,112,113,114,115,116,117].

A blood clone could influence the brain through circulating myeloid cells, platelets, cytokines, endothelial activation or coagulation without stable CNS engraftment. Decisive studies must connect clone-derived cells or products to a defined neural or behavioural phenotype; detecting a variant in bulk brain tissue is insufficient [106,114,117].

The most defensible model is modular: a driver changes clonal fitness and mature-cell behaviour; systemic exposures modify the clone; inflammatory, thrombotic or endothelial pathways affect vascular and barrier biology; and any brain effect depends on age, reserve, psychiatric liability and comorbidity. A clone may be harmful in one tissue and neutral or protective in another [2,109,112,118].

**Figure 2:**
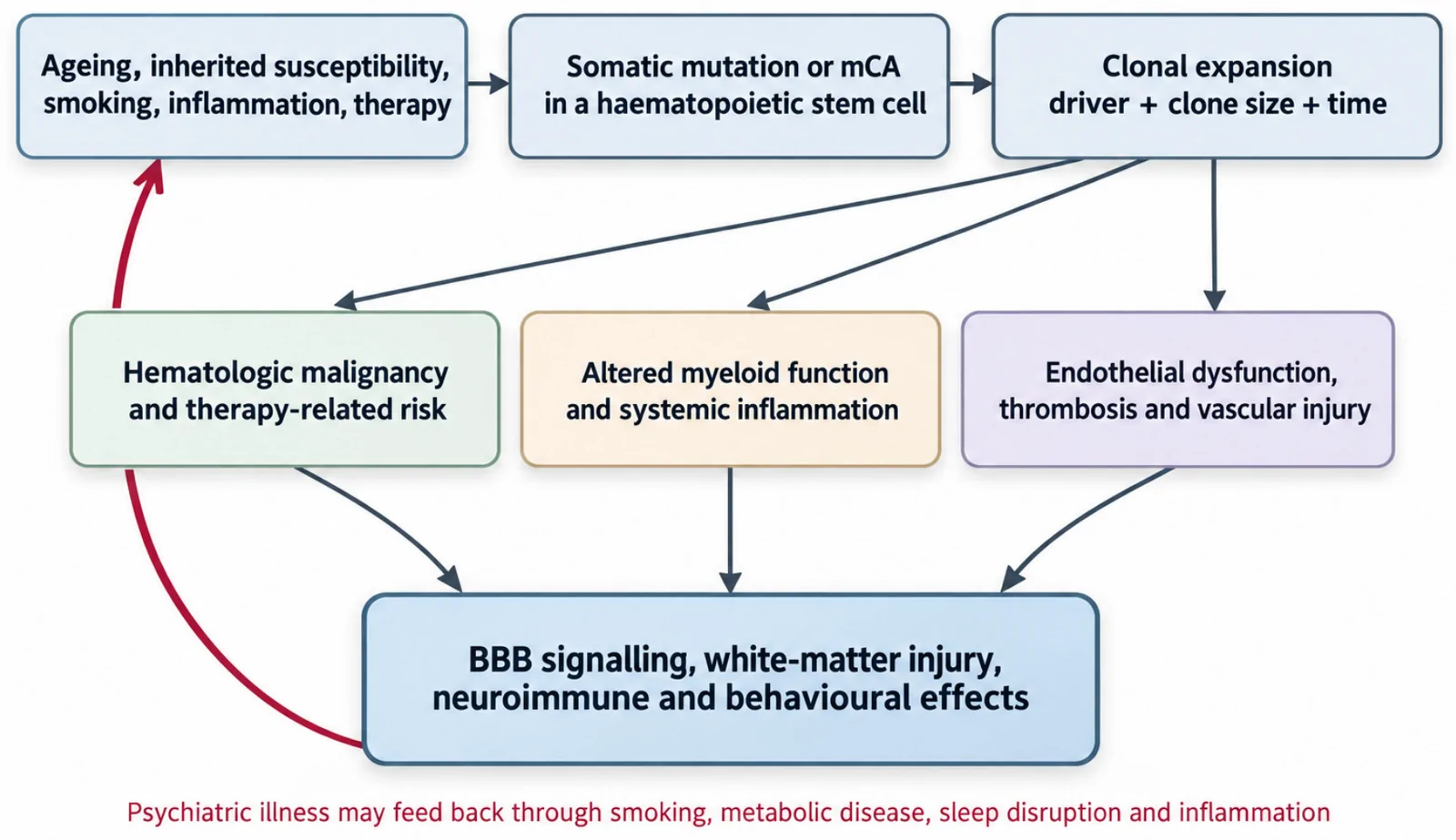
Conceptual bidirectional model linking somatic blood mosaicism, cancer susceptibility and psychiatric phenotypes. The arrows summarize plausible malignant, immune and vascular pathways and feedback from psychiatric illness or correlated exposures; they should not be interpreted as demonstrated causal paths.

Taken together, the available evidence supports biological plausibility for a bidirectional, multistep framework, but does not establish a causal pathway between somatic blood mosaicism and psychiatric disease. Figure 2 integrates the principal malignant, immune and vascular relationships, while Table 3 summarizes the proposed mechanisms and distinguishes indirect biological plausibility from direct psychiatric evidence.

## 5. Alternative Explanations and Causal Inference

Age is the dominant shared cause. It increases the prevalence and cell fraction of CH and mCAs while also increasing vascular disease, multimorbidity and late-life psychiatric vulnerability. Linear adjustment may be inadequate when survival and biobank participation select healthier older controls [3,40,81].

Smoking is associated with mLOY and specific CH patterns and independently affects psychiatric illness, cancer and cardiovascular disease. Pack-years, cessation timing and substance co-use are more informative than a binary covariate, particularly when substance-use disorder is an outcome [54,55,119].

Socioeconomic adversity, ancestry and environmental exposure can shape both clonality and psychiatric ascertainment through healthcare access, metabolic health, pollution, infection and survival. Cross-ancestry replication and detailed exposure measurement are necessary to separate these pathways [31,82,120,121,122,123].

Obesity, diabetes, atherosclerosis, kidney disease and chronic inflammation may be confounders, mediators or consequences. Adjustment choices should follow an explicit causal model because controlling a mediator can hide a pathway, while omitting a confounder can create one. Psychiatric illness may also influence clone growth through inflammation, sleep, inactivity or infection [100,102,124,125,126].

Psychotropic exposure remains poorly resolved. Medication can affect metabolic state, blood counts and medical surveillance, but cumulative dose, adherence, switching and illness severity are rarely measured well enough to separate treatment from indication. Active-comparator analyses, pharmacy-derived exposure and within-person clone-growth models would be more informative than ever-versus-never variables [13,18,19,119].

Cancer history requires mutation-aware analysis. DNA-damaging therapy selects *TP53*- and *PPM1D*-mutant clones, while cancer survivorship may also involve depression, pain, fatigue and substance exposure. Pooling treatment-selected clones with age-related *DNMT3A* or *TET2* CHIP can produce a clinically misleading average [34,65,69].

Clone presence, clone expansion and the process that generated a clone are different exposures. A baseline clone may mark ageing, inflammation, vascular injury, treatment selection or developmental mosaicism without causing psychiatric symptoms. Growth, acquisition of additional mutations and changing lineage output may matter more than binary detection.

All four causal models remain viable. Prospective ordering is strongest when clonality is measured before a first recorded diagnosis, but baseline clones can still follow years of subthreshold symptoms, smoking, metabolic disease or treatment. Cell fraction cannot date an event because it depends on driver fitness, lineage, age and selection [13,32,33,40,81,102].

**Figure 3:**
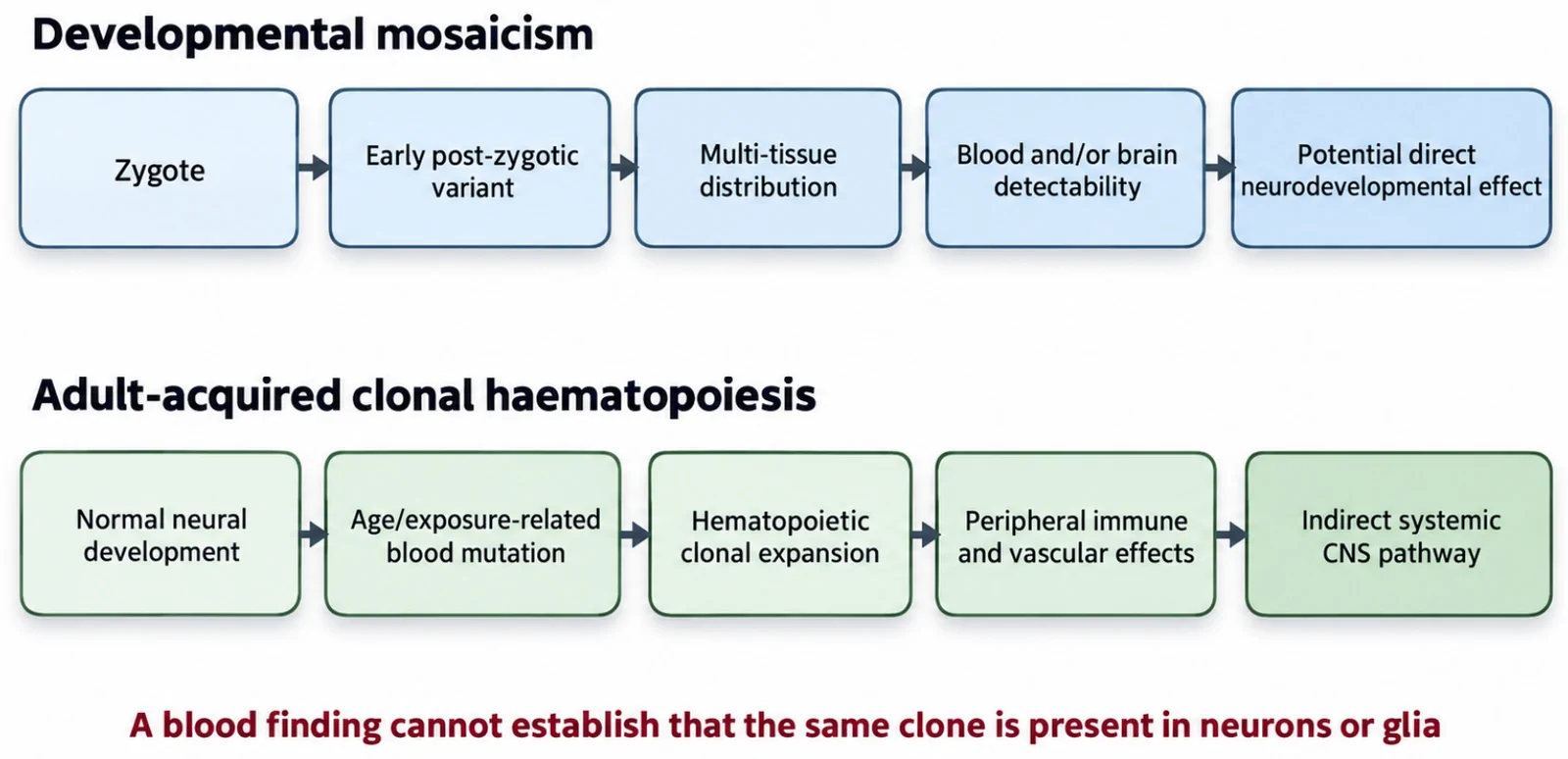
Developmental mosaicism versus adult-acquired clonal haematopoiesis. The timelines imply different tissues, mechanisms and causal questions. Detection of a variant in blood cannot by itself date the event or establish that the same clone is present in neurons or glia [20,32,33].

Developmental mosaicism and adult-acquired CH imply different mechanisms. Multi-tissue phylogeny can support an early event; repeated blood sequencing can capture acquisition and growth; and serial psychiatric, treatment, inflammatory, vascular and imaging measurements can test temporal mediation. No current psychiatric study combines these elements [14,16,17,20,32,33,41,81]. See Figure 3.

Time-varying confounding is especially important after psychiatric onset because medication, hospitalization, smoking changes and cytotoxic treatment may both follow illness and influence clonal selection. Larger clones can reflect duration or selection as well as biological dose [13,14,16].

Selection into biobanks, survival, sequencing and imaging can create collider bias when psychiatric and physical health influence participation. Studies limited to affected patients can also incur index-event bias. Inverse-probability weighting, negative controls and comparison with clinically recruited cohorts should supplement conventional adjustment [13,124,127].

Causal inference must therefore be triangulated across prospective cohorts, germline instruments, experimental models and human tissue studies. These designs answer different questions and carry different biases. Concordance would strengthen a driver-, tissue- and age-specific claim; disagreement should refine the model rather than be averaged into a generic causal effect [20,84,118,120,121,128,129]. See Table 4.

Somatic clonality may mark a subgroup with combined inflammatory, vascular, biological-ageing and oncologic vulnerability. Such a subgroup could cross diagnostic boundaries and differ in cognition, white-matter integrity, cardiometabolic burden or treatment course. This is a stratification hypothesis, not a diagnostic category [13,79,124].

Inflammatory phenotypes in depression and psychosis are neither specific nor universal. A CH-positive subgroup would need validation against multimodal phenotypes, including inflammatory proteins, metabolic state, vascular measures and MRI. No study has established a CH-defined psychiatric treatment-response class [95,128,130,131,132,133,134,135,136,137,138,139,140,141].

A clinically important reverse question is whether psychiatric patients with CH or mCAs have different rates of myeloid neoplasia, solid cancer, cardiovascular events or death. Established haematologic predictors—high-risk genes, multiple mutations, larger VAF, cytopenia and rapid expansion—should retain their usual meaning; psychiatric status could be tested as an effect modifier through exposure, inflammation or care disparities [5,6,142].

No study shows that psychiatric illness independently accelerates malignant transformation in clone carriers. Comparisons of clone-positive people with and without psychiatric disease require competing-risk models and should include intermediate outcomes such as clone growth, new co-mutations and persistent cytopenia [9,81,83,124,127].

Clinical surveillance should remain mutation- and blood-count-driven rather than diagnosis-driven. Unexplained cytopenia, progressive count changes or prior cytotoxic exposure warrant standard medical evaluation, but psychiatric diagnosis alone does not change current CHIP management [143,144].

## 6. Clinical Implications and Research Priorities

Routine psychiatric screening for CHIP or mCAs is not justified. Direct evidence is limited, common-outcome effect sizes are modest, action thresholds are undefined, and no intervention has improved psychiatric outcomes after clone detection. Testing may also generate incidental findings and low-value surveillance [13,145,146].

Testing is appropriate in research protocols or standard haematologic evaluation of unexplained cytopenia or prior cytotoxic exposure, not as diagnosis-based psychiatric screening. Clinical use would require analytical validity, independent replication, incremental prediction beyond routine risk factors, a clear management pathway and evidence that acting on the result improves outcomes [64,77,143,144,145,146,147]. See Table 5.

### 6.1. Minimum Reporting Standards

Genomic exposure definitions must be reproducible. WES-based CHIP at VAF at least 2% is not equivalent to error-corrected detection of smaller clones, and mCA calls depend on phasing, signal quality and cell-fraction thresholds. Studies should report gene lists, depth, validation, read requirements, VAF or cell-fraction distributions and threshold sensitivity analyses [27,30,44].

Psychiatric definitions require equal precision. Administrative diagnoses may miss onset, severity, recurrence, psychosis, treatment resistance and lifetime substance exposure; suicide is an outcome rather than a diagnosis. Clinically adjudicated or repeatedly recorded phenotypes should be used where possible [13,15].

Repeated sampling and clone growth are more informative than one binary blood measurement. Complete blood counts are needed to distinguish CHIP from CCUS or early myeloid disease, and brain mosaicism should not be inferred from blood. Developmental mosaicism, resident CNS myeloid mutation and adult CH require different sampling strategies [20,81,82,119,143,144,148,149,150,151,152].

Null and subgroup results should include carrier and event counts, confidence intervals, multiple-testing procedures, sex-specific analyses for sex-chromosome loss, and continuous as well as categorical clone fractions. Dramatic estimates from data-derived age groups require external replication because sparse events can dominate the result [14,15,16].

### 6.2. Research Priorities

The first priority is independent, ancestry-diverse replication with harmonized variant calling and repeated blood sampling. Baseline sequencing should capture driver, VAF and clonal complexity; phased SNP or genome methods should detect mCAs; and serial sampling should measure acquisition and growth alongside blood counts, germline susceptibility, smoking and cancer-treatment history [31,81,151].

Psychiatric assessment should establish diagnosis, onset, course, severity, cognition, suicide behaviour, substance exposure and cumulative medication history. Serial inflammatory, metabolic, vascular and MRI measures can then test mediation while cancer, myeloid neoplasia, cardiovascular events and mortality are adjudicated in parallel [13,56,137].

The central tests are whether clonality precedes psychiatric onset, whether illness or treatment accelerates clonal expansion, whether effects are lesion-, driver- and dose-specific, and whether inflammatory or vascular intermediates explain the association. Experiments should evaluate both harmful and protective effects across major drivers [90,91,109,125].

A staged programme should harmonize calls, phenotypes and covariates across existing cohorts; validate molecular and imaging signals in deeply phenotyped samples; recruit prospective clinical cohorts with serial sequencing and treatment data; and consider clinical pathways only after external validation demonstrates useful prediction and an intervention improves outcomes. Negative controls, family designs and suitable germline instruments can strengthen inference before trials [5,44,77,81,118,120,121,145,147].

## 7. Conclusions

Somatic blood mosaicism is established biology, but its psychiatric evidence base remains small. Prospective CHIP findings for depression and substance-use disorders rely mainly on one large cohort. mCA associations in schizophrenia, bipolar disorder and suicide are promising but heterogeneous and need independent clinical replication.

Inflammatory, myeloid and vascular pathways make indirect brain effects plausible, yet most mechanistic evidence comes from cardiovascular, stroke or neurodegenerative settings. Driver-specific and sometimes opposing effects argue against treating CHIP as one biological exposure. Blood detection also cannot establish developmental timing or neural presence.

Near-term studies should test both directions: whether clones contribute to psychiatric heterogeneity and whether illness, treatment or correlated exposures alter clonal evolution and subsequent medical risk. Without replicated temporal and mechanistic evidence, useful prediction and an effective intervention, somatic blood mosaicism remains a research hypothesis rather than a routine psychiatric biomarker.

## Figures and Tables

**Table 1:** Terminology and biological interpretation of somatic blood mosaicism.

Entity	Operational Definition	Typical Detection	Representative Alterations	Interpretive Caution and Sources
Clonal haematopoiesis	Expansion of blood cells derived from one stem or progenitor cell; may or may not have an identified driver.	Deep sequencing, WES/WGS, SNP arrays, lineage assays	SNVs, indels, mCAs, uncharacterized drivers	Umbrella category; clonality is not synonymous with malignancy [25,26]
CHIP	Recognized myeloid-neoplasm driver, commonly VAF ≥ 2%, without overt haematologic neoplasm or qualifying cytopenia.	Targeted NGS, WES/WGS	*DNMT3A*, *TET2*, *ASXL1*, *JAK2* p.V617F, *TP53*, *PPM1D*	Threshold is conventional and platform-dependent [24,27]
CCUS	Unexplained persistent cytopenia plus a myeloid-associated somatic clone, without diagnostic morphologic criteria for myeloid neoplasia.	CBC, marrow assessment and NGS	High-risk genes, co-mutations, larger VAF	Higher malignant risk than uncomplicated CHIP [29]
mCA	Large acquired chromosomal gain, deletion or copy-neutral LOH present in a fraction of blood cells.	Phased SNP array, WGS	Autosomal/X/Y losses or gains, CN-LOH	Not equivalent to mutation-defined CHIP; calling sensitivity varies [30,31]
Developmental mosaicism	Post-zygotic variant arising early enough to populate more than one tissue or cell lineage.	Multi-tissue deep sequencing, single-cell methods	SNVs, indels, CNVs, aneuploidy	Blood-brain concordance must be demonstrated, not assumed [32,33]
Therapy-related CH	Pre-existing or newly detected clones selected in the context of cytotoxic exposure.	Serial targeted NGS/WES	*TP53*, *PPM1D*, *CHEK2* and other DDR genes	Biology and prognosis differ from age-related DTA clones [34,35]

**Table 2:** Direct human studies of blood somatic mosaicism and psychiatric phenotypes. Study design and evidence category are shown explicitly to distinguish prospective associations from cross-sectional and post-mortem evidence. Temporal ordering, replication, and same-individual blood–brain concordance are also presented because effect estimates are not directly comparable across lesion classes or outcomes. Evidence categories describe study design and should not be interpreted as formal evidence grading.

Study and Design	Phenotype and Exposure	Principal Result	Timing and Diagnostic Sequence	Replication Status	Major Confounding and Tissue Interpretation	Evidence Category
Kimura et al. 2018 [17] Japan; 140 cases; 146 controls; brain subset	Suicide death; mLOY by *AMELY*/*AMELX* signal ratio	Blood mLOY OR 3.50; no case association in DLPFC	Post-mortem case–control design; acquisition relative to illness and death unknown	No independent replication; later study reported the opposite direction for male mLOY	Age, smoking and assay differences; same-individual neural clone was not demonstrated	Post-mortem case–control association
Hirata et al. 2018 [18] Japan; 146 SCZ cases; 214 controls	Schizophrenia; mLOY by *AMELY*/*AMELX* ratio	No prevalence difference; mLOY associated with illness duration within cases	Cross-sectional; illness preceded sampling, but clone acquisition was not dated	No independent replication	Age, smoking and illness duration; result is compatible with reverse causation	Cross-sectional case–control association
Jiang et al. 2023 [19] China; 271 SCZ cases; 171 controls	Schizophrenia; mLOY fraction by droplet digital PCR	Higher mosaic fraction, not higher mLOY prevalence; onset and duration associations	Cross-sectional; diagnosis preceded sampling; clone timing unknown	No independent replication	Age at onset, duration and age at sampling are difficult to separate; no brain testing	Cross-sectional case–control association
Chang et al. 2025 [14] China; 9715 SCZ cases; 28,822 controls; external datasets	Schizophrenia; filtered somatic CNV losses called with MoChA	Losses 1.00% versus 0.52%; OR 1.91; larger estimates at higher cell fraction	Case–control sampling; proposed developmental origin is inferential	Cross-cohort support, but not a matched clinical replication of identical events	Selection and calling thresholds; limited locus overlap, no same-person blood-brain concordance	Case–control association with cross-cohort support
Otsuka et al. 2025 [15] Japan; 773 cases; 177,740 controls; two cohorts	Suicide death; autosomal mCAs, mLOX and mLOY by SNP array	Autosomal mCA OR 1.78; losses 2.70; gains 2.23; female mLOX 4.00; male mLOY not Bonferroni significant	Post-mortem case–control design; acquisition relative to psychiatric course unknown	Consistent autosomal and mLOX signals across two study cohorts; no outside replication	Lifetime illness, smoking, adversity and treatment; no same-person brain concordance	Post-mortem case–control association across two cohorts
Uchiyama et al. 2026 [16] Japan; 2470 SCZ; 3732 BD; 177,773 controls	Schizophrenia and bipolar disorder; blood mCAs with cell-fraction thresholds and PRS	Autosomal mCAs OR 1.78 and 1.41; young-age OR 7.01 and 4.01; cell-fraction gradient	Case–control design; timing of mCA acquisition relative to diagnosis unknown	No independent clinical replication	Sparse young-age events, subgroup selection, survival and biobank participation; no brain concordance	Large case–control association
Yu et al. 2026 [13] UK Biobank; *n* = 360,451; prospective	Thirteen incident psychiatric outcomes; 74-gene WES CHIP; imaging subset	Any disorder HR 1.15; supported depression and substance-use associations; white-matter differences	Blood measurement preceded recorded diagnosis; earlier subthreshold illness and exposures remain possible	Single discovery cohort; dose, gene and imaging analyses are internal, not independent replication	Age, smoking, cardiometabolic health, SUD-related exposure, selection and imaging-subset bias; no neural clone test	Prospective cohort association

Abbreviations: OR, odds ratio; HR, hazard ratio; DLPFC, dorsolateral prefrontal cortex; PRS, polygenic risk score; SCZ, schizophrenia; BD, bipolar disorder.

**Table 3:** Candidate mechanisms connecting haematopoietic clones to psychiatric phenotypes, with the evidence level distinguished from direct psychiatric evidence.

Mechanism	Biological Pathway	Evidence Base	Psychiatric Relevance	Evidence Level and Principal Uncertainty
Inflammasome and cytokine signalling	TET2 or related mutant myeloid cells alter IL-1beta NLRP3 and IL-6 pathways.	Human genetics, cardiovascular cohorts and mouse models [85,86,88]	Transdiagnostic inflammatory subgroups and depression risk	Indirect human + experimental evidence; no direct psychiatric mediation test.
Myeloid cell reprogramming	Driver-specific monocyte macrophage transcription, metabolism, phagocytosis and trafficking.	Single-cell and experimental disease models [37,106,117]	Peripheral-to-central immune signalling	Experimental/single-cell evidence; psychiatric relevance remains mechanistic inference and may reverse by tissue context.
Vascular injury and thrombosis	Atherosclerosis, endothelial dysfunction, thrombosis and cerebrovascular events.	Large human cohorts plus causal mouse studies [83,90,104]	Late-life mood, cognitive and white-matter phenotypes	Indirect human + causal animal evidence; psychiatric mediation and competing risk are insufficiently tested.
BBB and neurovascular signalling	Systemic inflammation changes endothelial junctions, permeability and neurovascular coupling.	Stress and inflammatory models; limited human tissue evidence [110,111]	Mood and psychosis-relevant neural environments	Experimental plausibility; limited direct CH evidence in psychiatric populations.
Mutated CNS-associated myeloid cells	Circulating clones contribute to microglia-like populations or alter resident myeloid states.	Alzheimer cohorts and mouse models [112,114,116]	Potential direct CNS immune effect	Neurodegenerative human/animal evidence; context-dependent and may be protective or harmful.
Shared biological ageing	Ageing independently increases clonality, inflammation, vascular injury and psychiatric vulnerability.	Consistent epidemiology and longitudinal clonal studies [40,81]	Broadest alternative explanation	Epidemiologic alternative explanation; strong confounding potential and biological age is imperfectly measured.

**Table 4:** Factors that may influence observed associations between somatic blood mosaicism and psychiatric phenotypes. The potential epidemiological role of each factor is context-dependent and may vary according to temporal ordering, study design, and the causal model under consideration.

Factor	Relationship with Somatic Blood Mosaicism/Clonal Haematopoiesis	Relationship with Psychiatric Phenotypes	Potential Epidemiological Role	References
Age	Increasing age is strongly associated with higher prevalence and burden of CH and mCAs	Age influences psychiatric incidence, multimorbidity, vascular disease, and cognitive outcomes	Major confounder	[3,40,81]
Smoking	Associated with mLOY and selected patterns of clonal haematopoiesis	Associated with psychiatric morbidity and substance-use phenotypes	Confounder; potential contributor to reverse causation	[54,55,119]
Ancestry and germline susceptibility	Influence susceptibility to CH, mCA distribution, and clonal fitness	May correlate with population structure, environmental exposure, healthcare access, and ascertainment	Confounder/population-structure factor	[31,82,120,121]
Socioeconomic and environmental exposures	May influence clonal selection through infection, pollution, metabolic stress, and related exposures	May influence psychiatric risk, healthcare access, diagnosis, and treatment	Potential confounder	[119,122,123]
Obesity and metabolic disease	Metabolic and inflammatory states may influence haematopoietic stem-cell fitness and clonal expansion	May precede, accompany, or follow psychiatric illness and its treatment	Confounder, mediator, or consequence	[100,124,125,126]
Chronic inflammation	May alter haematopoietic stem-cell fitness and promote expansion of selected clones	Associated with inflammatory subgroups across several psychiatric disorders	Confounder, mediator, or component of bidirectional feedback	[95,96,97,98,99,100,101,102,128,129,130,131,132,133,134,135,136]
Psychotropic treatment	Direct effects on clonal evolution remain insufficiently characterized; treatment may affect metabolic state, blood counts, and medical surveillance	Closely linked to illness severity, duration, and treatment history	Potential confounder or consequence; susceptible to indication bias	[13,18,19,119]
Cancer and cytotoxic treatment	Chemotherapy and radiotherapy can select treatment-associated clones, particularly involving DNA-damage-response genes	Cancer diagnosis, treatment, and survivorship may influence psychiatric outcomes and healthcare exposure	Confounder; source of treatment-related clonal selection	[34,35,64,65,66,67,68,69]
Cardiovascular and cerebrovascular disease	Associated with CH and may also occur downstream of clone-related inflammatory or thrombotic pathways	May influence mood, cognition, and white-matter phenotypes	Confounder or mediator, depending on temporal ordering	[83,103,104,105,106,107,108,109,124,127]
Psychiatric illness duration and cumulative exposure	Longer disease duration may coincide with cumulative smoking, metabolic disease, inflammation, treatment, or other selective pressures on clones	Reflects cumulative psychiatric disease and treatment burden	Potential source of reverse causation	[18,19,119]
Biobank participation, survival, and sequencing/imaging selection	Determine which clone carriers enter and remain in analytic cohorts	Psychiatric and physical health may influence participation, survival, and availability of genomic or imaging data	Selection bias/collider bias	[13,124,127]

**Table 5:** Evidence required before psychiatric testing for somatic clonality could become actionable.

Domain	Current Position	Missing Evidence	Required Design	Potential Implication
Analytical validity	Established for research sequencing and array pipelines but threshold-sensitive.	Cross-platform concordance at low VAF and low mCA cell fraction.	Blinded technical replication and orthogonal validation [27,44]	Reliable classification of exposure
Clinical validity	Associations reported for depression, substance use, schizophrenia, bipolar disorder and suicide.	Independent replication with granular diagnosis and treatment data.	Prospective multi-cohort validation [13,16]	Credible phenotype-specific estimates
Causal validity	Dose gradients and plausible immune vascular mechanisms.	Temporal ordering, mediation and exclusion of shared causes.	Repeated sequencing plus causal mediation and negative controls [81,102]	Distinguish cause marker and consequence
Incremental prediction	Not established beyond age, blood counts, smoking and multimorbidity.	Calibration, discrimination and decision-curve benefit.	Externally validated risk models [5,77]	Identify who benefits from action
Clinical utility	No psychiatric management pathway or outcome-improving intervention.	Benefit-harm balance, patient-reported outcomes and cost-effectiveness.	Pragmatic mutation-stratified trials [145,146]	Evidence-based selective testing
Implementation and ethics	Incidental findings and multidisciplinary referrals are common.	Communication standards, equity and follow-up capacity.	CH clinic implementation studies [144,147]	Safe and equitable care

## Data Availability

No new data were created or analyzed in this study. Data sharing is not applicable to this article.
